# Hub Promiscuity in Protein-Protein Interaction Networks

**DOI:** 10.3390/ijms11041930

**Published:** 2010-04-26

**Authors:** Ashwini Patil, Kengo Kinoshita, Haruki Nakamura

**Affiliations:** 1 Human Genome Center, Institute of Medical Science, The University of Tokyo, 4-6-1 Shirokane-dai, Minato-ku, Tokyo 108-8639, Japan; E-Mail: ashwini@hgc.jp; 2 Graduate School of Information Sciences, Tohoku University, 6-3-09, Aramaki-aza-aoba, Aoba-ku, Miyagi, 982-0036, Japan; E-Mail: kengo@ecei.tohoku.ac.jp; 3 Bioinformatics Research and Development, Japan Science and Technology Corporation, 4-1-8 Honcho, Kawaguchi, Saitama 332-0012, Japan; 4 Institute for Protein Research, Osaka University, 3–2 Yamadaoka, Suita, Osaka 565-0871, Japan

**Keywords:** protein-protein interactions, interaction networks, hubs, promiscuous binding

## Abstract

Hubs are proteins with a large number of interactions in a protein-protein interaction network. They are the principal agents in the interaction network and affect its function and stability. Their specific recognition of many different protein partners is of great interest from the structural viewpoint. Over the last few years, the structural properties of hubs have been extensively studied. We review the currently known features that are particular to hubs, possibly affecting their binding ability. Specifically, we look at the levels of intrinsic disorder, surface charge and domain distribution in hubs, as compared to non-hubs, along with differences in their functional domains.

## 1. Introduction

Diseases in living organisms are usually the result of a disruption in the function of one or more proteins in its cells. Proteins function through their interactions with other proteins, small molecules, DNA and RNA. In order to understand the role of a protein in any cellular mechanism, it is critical to identify its interactions. As a result, the identification and mapping of protein-protein interactions has recently received a lot of attention. Small-scale experiments studying the interactions of a single or a small set of proteins are informative but do not provide a global perspective of the inter-relationships between proteins. Towards this end, several groups have performed large-scale, or high-throughput, experiments in different organisms [1–9]. The resulting interaction networks, though by no means exhaustive, have provided a starting point for systems level studies of proteins and their interactions. With the help of recent advances in complex network theory and its application to biological networks [10], the study of protein-protein interaction networks has come of age.

**Figure 1.**
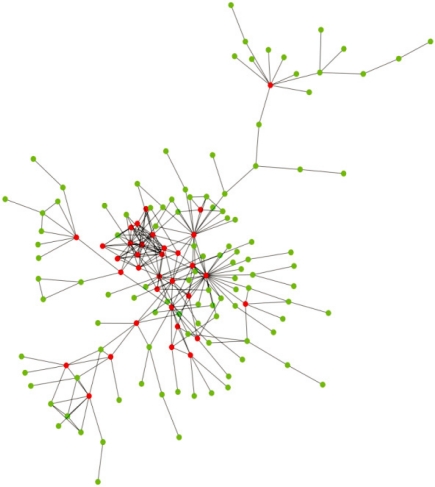
Partial human protein-protein interaction network showing scale-free topology. Hubs (proteins with 5 or more interactions) and non-hubs are denoted by red and green nodes, respectively. Interactions are shown by the black links between the nodes.

Protein-protein interaction networks in all species have properties similar to other complex networks, like the World Wide Web [10]. These networks are known as scale-free networks and are characterized by a power-law degree distribution [11]. This means that most of the proteins (nodes of the network) share a few interactions (have a small number of links between them), whereas, a small percentage of proteins interact with a disproportionately large number of other proteins (have a large number of links in the network). Such proteins, or nodes, with a large number of interactions (links) are called hubs. Figure 1 illustrates this concept with the help of a partial human protein-protein interaction network.

This network topology provides a high level of robustness to the network since the failure of a few random nodes does not affect the function of the network drastically. However, it also makes the network more vulnerable to defects in the hubs, which can cause a large part of the network to fail due to their large number of connections. In interaction networks, as well as other biological networks, the deletion of a hub has been shown to be lethal to the organism [12]. It is clear that hubs are central to the normal function and stability of the protein-protein interaction network in any organism. Several well-known and extensively studied proteins that are implicated in diseases are hubs. Examples include p53, p21, p27, BRCA1, kalirin, ubiquitin, calmodulin and many others which play central roles in various cellular mechanisms. This makes them important and interesting subjects for further study.

Hubs in protein-protein interaction networks have been classified into two main categories - transient (participate in a single interaction at a time) and obligate (participate in multiple simultaneous interactions). However, they have been termed differently by different groups as date and party, sociable and non-sociable and singlish interface and multi-interface, though these terms are not always equivalent. Date and party hubs were first identified by Han *et al*. using coexpression correlation coefficients of hubs with their partner proteins [13]. Date hubs were denoted as transient due to their low average coexpression correlation with their interaction partners, while party hubs were designated as obligate because of their high coexpression correlation. However, this classification is still highly debated [14–16]. Higurashi *et al*. divided hubs into stable, sociable and non-sociable hubs using protein structure data and the number of interaction interfaces of the hub [17]. They considered hubs that were part of stable complexes as stable or obligate. They further classified the remaining hubs into sociable (with three or more binding sites) or non-sociable (only one binding site). Sociable hubs were considered as transient. Kim *et al*. also used the number of interaction interfaces of hubs as obtained from protein structure data to define singlish-interface and multi-interface hubs [18]. Singlish interface hubs were those with one or two binding interfaces, while multi-interface were those with three or more interfaces. In this study, singlish interface and multi-interface hubs were considered to be transient and obligate, respectively. Thus, the classification of hubs into transient and obligate has been contentious with different groups using different criteria to identify them.

One of things that is immediately apparent from the definition of hubs in protein-protein interaction networks is their ability to recognize and bind to many other proteins. Interactions in proteins are mediated by the recognition of distinct binding regions by the protein on the surface of its interaction partner. Such molecular recognition must be specific enough and of sufficient affinity for the interaction to take place. The binding promiscuity of hubs raises the question of their ability to recognize, with required specificity, the binding regions in the partner proteins. Thus, the central question in the operation of a hub protein is: does a hub protein have any special structural characteristics that facilitate the recognition of multiple interaction interfaces in other proteins? In this review, we discuss possible explanations for this question by exploring the structural properties, as well as other features, of hubs that have identified so far by us and others.

## 2. Structural Characteristics of Hub Proteins

In order for a protein to recognize and bind several other proteins, it is imperative for it to have some structural characteristics that aid this process. The structural properties of hubs, as compared to non-hubs, have been extensively studied in the last couple of years. We discuss each of these in detail below. The definition of a hub for the purposes of such analyses has been quite varied, with some studies defining hubs as proteins with five or more interactions [12,19], while others defining them as proteins with 10 or more interactions [20]. Other criteria, like a floating cutoff for the number of interactions [21] and subgraph connectivity [22] have also been used for the identification of hub proteins. However, the criterion of five or more interactions has proved to be a robust one [22] and has been used in the identification of most of the characteristics described below.

### 2.1. Intrinsic Disorder

Structural flexibility or the ability of a protein to fold into an ensemble of conformations is one the most significant factors affecting its binding ability. This flexibility allows the protein to adopt different structural conformations when bound to different targets. Structural flexibility in a protein can either be local or global. Local flexibility is manifested in the form of small flexible loops or coils in a folded protein. These loops or coils are flexible parts of a folded globular protein that take on different conformations when binding different targets. On the other hand, global flexibility is the result of the presence of large intrinsically disordered or unstructured regions in the protein. Disordered regions are large unfolded regions in a protein that have no tertiary structure and little or no secondary structure [23,24]. Inspite of their apparent unfolded state under physiological conditions, proteins with disordered regions are surprisingly common in organisms across all orders of life, with their prevalence increasing with the complexity of the organism [23]. 33% of eukaryotic proteins contain disordered regions greater than 30 residues in length [25]. Disordered regions are prevalent in several signal transduction proteins as well as those implicated in cancer [26].

The hypothesis that hubs may acquire the flexibility they need through the presence of disordered regions was first proposed by Dunker *et al*., where they categorized hubs based on their levels of disorder and those of their interaction partners, citing several examples of hubs with large disordered regions [27]. Further testing this hypothesis, we performed a large scale analysis using filtered interaction datasets across several species and studied the prevalence of loops/coils and disordered regions in hubs [19]. In this study, loops/coils were the small flexible regions of the protein that had a tertiary structure but lacked a secondary structure (*i.e.*, were not part of an alpha helix or a beta sheet). On the other hand, regions with more than 30 consecutive residues which were either predicted as disordered or had missing electron density (*i.e.*, lacking tertiary structure) were considered as disordered regions. It was found that hubs had a higher percentage of disordered residues than non-hubs. Hubs also had fewer loops/coils than non-hubs. Though this does not necessarily diminish the role of loops/coils or small flexible regions in hubs, it indicates that disordered regions play an important role in the way hubs function. These findings were later supported by several other groups [20,28,29]. However, there is no direct correlation between the number of interactions of a hub and the percentage of disordered residues present [19] complicating the exact role played by disordered regions in promiscuous binding.

**Figure 2.**
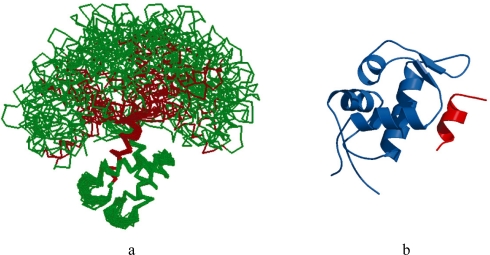
(**a**) NMR solution structure of Calmodulin showing the relative motion of one Ca^2+^ binding domain (green) with respect to the other using the flexibility of the central disordered region (red) (PDB ID: 1DMO). (**b**) X-RAY Crystal structure of a small fragment of the N-terminal disordered region of p53 (red) bound to MDM2 (blue) (PDB ID: 1YCQ).

The disordered region in the hub may be present in one of two forms. Firstly, it may act as a flexible linker that connects two ordered domains allowing them unrestricted movement with respect to each other. For instance, Ubiquitin-conjugating Enzyme (Ubc1), an E2 ubiquitin ligase, is a hub with a 22 residue disordered region that acts as a flexible linker [30]. Similarly, the flexible linker in Calmodulin is a 36 residue disordered region that connects its two Ca^2+^-binding domains allowing it to bind several targets [31] (Figure 2a). Secondly, the disordered region may itself be the binding region as in the case of the transcription factor and tumor suppressor, p53, which binds to the E3 ubiquitin ligase, MDM2, using its N-terminal disordered region. This region undergoes a disorder-to-order transition when it binds to MDM2 [32] (Figure 2b). Similarly, in the small cyclin-dependent kinase inhibitors p21^Waf1/Cip1/Sdi1/Cap20^ and p27^Kip1^, the multi-specific binding site is located in N-terminal disordered region that undergoes an order-to-disorder transition on binding the cyclin-CDK complexes [33,34]. In some cases, the disordered region may act as both a linker and a binding domain as seen in the tumor suppressor Breast cancer type-1 susceptibility protein (BRCA1). BRCA1 has a large central disordered region of approximately 1,500 residues which not only binds DNA and several proteins, but also acts as a flexible linker between its N-terminal RING domain and two C-terminal BRCT domains [35].

Disordered regions provide several advantages to hubs. Disordered regions not only provide global flexibility but can also undergo folding induced by the recognition of, and binding to, their multiple targets [24,36]. It has been proposed that the presence of disordered domains allows for faster binding of a protein to its target with high specificity through the “fly-casting mechanism”. In this mechanism, an unfolded protein weakly binds its target over large distances and then folds as it approaches the interaction site to bind with high affinity [37,38], with the disordered regions protruding into, or interwinding with, the binding interface of the target [39]. It has also been suggested that the interactions resulting from the fly-casting mechanism may be of high affinity with the increasing levels of disorder used to modulate the binding affinity [40]. Proteins with disordered regions can also be more tightly regulated because they have rapid turnover times due to their susceptibility to proteolytic degradation [23,41]. Interestingly, it has also been shown that the interaction partners of hubs have higher levels of intrinsic disorder than expected [27,42], and that proteins with disordered regions preferentially interact with each other, especially in the case of non-hubs [43]. The identification, properties and advantages of disordered regions in proteins have been recently discussed in depth by Dunker *et al*. [44].

Studying the different types of hubs, Singh *et al*. found that transient, or date, hubs have higher levels of intrinsic disorder than obligate, or party, hubs [45]. However, these results should be treated with caution since the initial classification into date and party hubs by Han *et al.* is still disputed. More reliable results were obtained by Higurashi *et al*., who find that though sociable (transient) hubs do not have a higher level of disorder at their interfaces as compared to non-sociable proteins, they do have a greater overall structural flexibility [17]. It would be interesting to find the levels of disorder in the entire proteins in the sociable and non-sociable groups for a better understanding of the role of disordered regions in the global flexibility of these proteins. Kim *et al.*, showed that singlish interface hubs (transient) have higher levels of disorder than multi-interface hubs (obligate) [42]. Thus, the role of intrinsic disorder in transient and obligate hubs is still not clear. Further studies await a better classification technique for the clear identification of transient and obligate hubs.

### 2.2. Surface Charge

**Figure 3.**
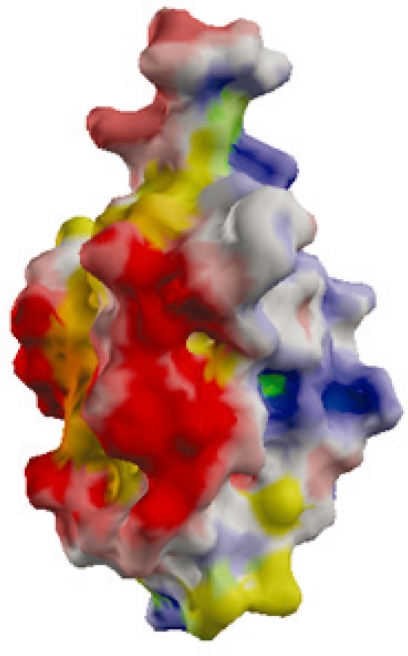
Surface electrostatic potential of Cofilin (obtained from eF-site [54]) (PDB ID: 1QPV). Negative potential is indicated in red, positive potential in blue and hydropathy in yellow.

Several small hubs (size less than 300 residues), though not all, have very few or no disordered residues [19]. Therefore, the flexibility afforded by disordered residues, does not explain the multiple recognition capabilities of such hubs. Examples include Ubiquitin, Ferredoxin, Ras, and other small GTPases. Figure 3 shows the surface potentials of the small actin-binding hub, Cofilin, which has a highly charged surface but lacks large disordered regions. A study of the charged residues on the surfaces of such small hubs shows that they have highly charged surfaces as compared to large, disorder containing hubs indicating their possible involvement in promiscuous binding [19]. A further analysis of the charged residues on the surfaces of hubs shows that most of the charged residues, except Arginine, are not located at the interface, but are distributed over the exposed surface of the protein [46]. Together these findings point to an indirect role of the charged residues in the promiscuous binding of small hubs. Electrostatic interactions of charged surface residues are known to affect the specificity of binding [47], complex stabilization [48] and promiscuous binding [49]. Specifically, these residues facilitate binding through the reduction of electrostatic binding free energy, via intra-molecular interactions within the hub or its partner protein, and long-range inter-molecular electrostatic interactions. It is known that the formation of a protein complex is preceded by an encounter complex which involves few specific interactions between the proteins and multiple changes in their relative orientation. It has been recently proposed that the charged surface residues outside the interface may promote the formation of such a dynamic encounter complex through long-range electrostatic interactions facilitating the formation of a final productive complex [50]. Additionally, the interfaces of hubs tend to be enriched in residues that facilitate the formation of multiple types of favorable interactions, like Arginine (Arg), Tyrosine (Tyr), Histidine (His) and Methionine (Met) [46]. Arg and Tyr are known interface hotspots with the ability to participate in several favorable interactions [51]. His is also an interface hotspot possibly due to its ability to form hydrogen bonds [52]. Met, like Arg, is a good anchor residue with a flexible side chain [53]. The specific enrichment of these residues at the interfaces of hubs may further enhance their ability to form multiple interactions.

The relationship between surface charge and intrinsic disorder is a complementary one, with surface charge acting primarily in small hubs and disorder acting in large hubs [19]. The role of charged surface residues in large hubs is yet to be studied and promises to lead to further interesting insights into the relationship between surface charge and intrinsic disorder in the molecular recognition capability of hubs.

### 2.3. Domain Distribution and Enrichment

Promiscuity in proteins can either be through the use of a single interaction site in order to bind multiple partners or through the use of multiple interaction sites for each different interaction partner. Humphris *et al*. studied the structures of several small hubs in complexes in order to design a multi-specific interface [55]. They found two groups of hubs – those that use a single interface to bind their interaction partners (Importin-beta, Elastase, Thioredoxin), and those that use multiple, and sometimes overlapping, interfaces for binding (Ubiquitin, Ras, Cdc42), indicating that multiple interfaces exist even in small hubs with only a single domain. Binding interfaces can further be partitioned into affinity-defining residues and specificity-defining residues, as seen in the case of Calmodulin [56]. Ordered domains in a protein often host the binding sites. Therefore, these domains play an important role in interaction formation of proteins with or without the aid of long disordered regions. A study of the prevalence of multi-domain and single domain architectures in hubs and non-hubs shows that hubs have a greater tendency for multi-domain architectures with the number of interactions of a hub positively correlated with the number of distinct domains in the protein [57]. The presence of multiple domains potentially provides hubs with multiple molecular recognition sites, thus leading to multiple interactions. Further scrutiny of the nature of ordered domains, using their Pfam annotations, indicates that hubs are enriched in kinase domains and adaptor domains, like SH2 and SH3 [57], both of which are reusable and promiscuous, providing hubs with multi-specific recognition ability [58]. Clearly, not only the number of domains, but also the nature of domains in hubs is significant in their function.

A comparison of the levels of disorder in single domain and multi-domain hubs shows that, on average, single domain hubs have a greater fraction of disordered residues than multi-domain hubs [57]. Similar results were obtained by Kim *et al*., who found singlish-interface hubs to have higher levels of intrinsic disorder as compared to multi-interface hubs [42]. This signifies a greater role for disordered regions in the absence of multi-domain architectures in hubs. However, it is still unclear how disordered regions may be functioning in proteins with single *versus* multi-domain architectures.

## 3. Other Perspectives

The promiscuous nature of proteins has been studied from other perspectives as well. Tsai *et al*. suggest that the promiscuity of hubs is not merely the result of a single protein binding many others, but different forms of the protein obtained from a single gene [59]. Briefly, they propose several mechanisms like alternative splicing, post-translational modification, allostery and combinatorial domain linkage which result in either, different domain architectures, different conformations or different specificity for the hub protein. Alternative splicing has been known to affect the structure of proteins and their binding sites through the exclusion of exons [60]. Conversely, it has also been shown that alternatively spliced regions in genes are often manifested as disordered regions in the protein in order to minimize its impact on the protein structure [61]. Thus, it is still not clear what the primary means of promiscuity due to alternative splicing is – is it change in domain architecture, change in the fraction of intrinsic disorder, or change in binding site? Most likely, it is all of these factors acting in concert with each other. Assuming that alternatively spliced isoforms help the promiscuity of hubs, it can also be hypothesized that hubs would have a greater tendency for alternative splicing than non-hubs, and therefore more isoforms resulting in more binding sites or conformations. This needs to be clarified by further study. Similar to the case of alternative splicing, sites associated with post-translational modification like phosphorylation [62] and ubiquitination [63] tend to have high levels of disorder calling into question the primary means of promiscuity. It would be interesting to find out if hubs are more often targets of post-translational modification or have a greater tendency towards allostery as compared to non-hubs.

**Figure 4.**
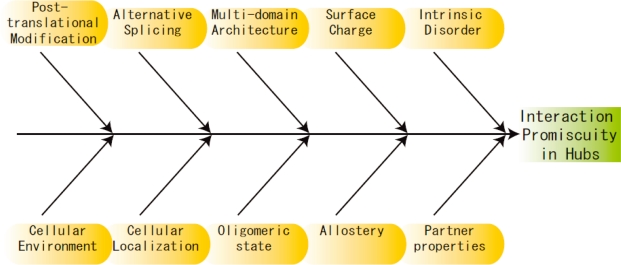
Ishikawa (Fishbone) diagram representing the characteristics affecting the interaction promiscuity in hub proteins.

Other possible causes of protein promiscuity have been reviewed by Nobeli *et al*. [64]. These authors consider protein flexibility as one of the primary causes of protein promiscuity. However, their discussion focuses on the flexibility of loops in the protein, rather than disordered regions. The role of flexibility in the form of loops and coils in hubs is important and needs further attention. For instance, the C-terminal of Ubiquitin shows conformational flexibility when observed using NMR Spectroscopy [65]. This could provide a further means of binding multiple proteins. Apart from the features discussed above, Nobeli *et al*., also review the roles of the oligomeric state of the protein, the phenomenon of partial recognition of the interaction partner, the presence of multiple interaction residues in a single interaction site, the size and complexity of the interaction partners and the role of the solvent in the interaction [64]. Without a doubt these characteristics and other environmental features, like cellular localization of the protein, the concentration of its interaction partners, the conditions in the cell also affect the binding ability of hubs. Figure 4 provides an overview of the many characteristics (some already studied, and others needing clarification) used by hubs to achieve promiscuity.

## 4. Future Directions and Challenges

We discussed numerous characteristics that affect the ability of hubs in protein-protein interaction networks to recognize and bind multiple partners. We primarily focused on the role of intrinsic disorder in the protein structure, the surface charge and the domain architecture of hubs. We also briefly touched on other characteristics that are important in the functioning of a hub. There are several questions in this regard that need to be addressed, the most pertinent being, which if not all of these features, affect the binding ability of hubs and to what extent. There is also a need to further elucidate the means through which disordered regions affect the binding ability of a protein. The role of alternative splicing and post-translational modification needs to be addressed in more detail as also the extent of their impact.

We are just beginning to understand some of the mechanisms that lead to multi-specificity in the binding of hub proteins. Other mechanisms await identification. It will be interesting to see how the study of promiscuity in hub proteins progresses and where it takes us with respect to understanding the many mechanisms through which proteins execute their functions.
