# Correction: Patil, A. *et al*. Hub Promiscuity in Protein-Protein Interaction Networks. *Int. J. Mol. Sci.* 2010, *11*, 1930-1943

**DOI:** 10.3390/ijms11082791

**Published:** 2010-07-27

**Authors:** Ashwini Patil, Kengo Kinoshita, Haruki Nakamura

**Affiliations:** 1 Human Genome Center, Institute of Medical Science, The University of Tokyo, 4-6-1 Shirokane-dai, Minato-ku, Tokyo 108-8639, Japan; E-Mail: ashwini@hgc.jp; 2 Graduate School of Information Sciences, Tohoku University, 6-3-09, Aramaki-aza-aoba, Aoba-ku, Miyagi, 982-0036, Japan; E-Mail: kengo@ecei.tohoku.ac.jp; 3 Bioinformatics Research and Development, Japan Science and Technology Corporation, 4-1-8 Honcho, Kawaguchi, Saitama 332-0012, Japan; 4 Institute for Protein Research, Osaka University, 3-2 Yamadaoka, Suita, Osaka 565-0871, Japan

We would like to change reference 56 on page 1942 of the article [1] from:

56. Fromer, M.; Yanover, C.; Linial, M. Design of multispecific protein sequences using probabilistic graphical modeling. *Proteins* **2009**, *78*, 530–547.

To the correct one as follows:

56. Fromer, M.; Shifman, J.M. Tradeoff Between Stability and Multispecificity in the Design of Promiscuous Proteins. *PLoS Comput. Biol.* **2009**, *5*, e1000627.

We apologize for any inconvenience brought to the readers.
